# Flurpiridaz F18: a new era in nuclear cardiac imaging – the first FDA-approved radiotracer in 30 years

**DOI:** 10.1097/MS9.0000000000004032

**Published:** 2025-10-07

**Authors:** Biruk Demisse Ayalew, Nawal Khaliq, Ali Dheyaa Marsool, Aayat Kashif, Ekram Muhammedasrar Ahmedelhadi, Firdowes Feki Seid, Muhammad Areeb Ul-Haq, Agazi Teweldeberhan, David N. Smith

**Affiliations:** a Department of Internal Medicine, St. Paul’s Hospital Millennium Medical College, Addis Ababa, Ethiopia; b Department of Internal Medicine, Internal Medicine, Dow University of Health Sciences (DUHS), Karachi, Pakistan; c School of Medicine, University of Baghdad/ Al-Kindy College of Medicine, Iraq; d Innah Sindh Medical University, Karachi; e Department of Internal Medicine, Ethio Tebib General Hospital; f Allama Iqbal Medical College, Lahore; g Intermediate Hospital, Oshakati, Namibia; h Yale University School of Medicine

## Introduction

27 September 2024 is marked as a significant milestone for nuclear medicine with the approval of Flurpiridaz F-18 to be used as a radiotracer for cardiac nuclear imaging. After almost 30 years of extensive research, this radiotracer can now be used for the diagnosis of multiple cardiovascular pathologies, mainly coronary artery disease (CAD) in adults[1].

As a non-invasive procedure utilizing radiotracers and single-photon emission computed tomography (SPECT) or positron emission tomography (PET) scanners, nuclear cardiac imaging scans gamma rays released by radiotracers in the heart. They provide an accurate alternative to the assessment of myocardial perfusion, viability, and functionality. This procedure can also identify early-stage CAD, way before symptoms are seen. Quantitative and objective measurements, such as ejection fraction and wall motion, are important for monitoring disease progression, while its ability to identify ischemic and non-viable areas can be used for risk stratification and decision-making. All the above qualities make nuclear cardiac imaging a valuable method for the diagnosis and treatment decision-making for heart failure and CAD[2].

Flurpiridaz provides superior sensitivity and specificity that is even more vital for patients where the diagnosis of CAD can be difficult, including obese patients. With a half-life of 110 minutes, it provides an advantage of centralized production and wider availability over other PET tracers that need on-site cyclotrons. Additionally, with a reduced radiation dose and a quicker scan, it gives a safer and better patient experience^[1,3]^.

No AI was used for research and manuscript development[4].

## Background

Flurpiridaz F-18 is a radiopharmaceutical drug that is used for PET myocardial perfusion imaging (MPI). MPI is an imaging process that is used in patients with known or suspected CAD. Hence, Flurpiridaz F-18 is a radioactive diagnostic agent, a drug that is used for an imaging test that shows how well blood flows through the heart muscle[1].

The approval of Flurpiridaz F-18 for PET in CAD patients was mainly influenced by the work of results of a second phase 3 prospective multicenter clinical trial and prior trials[5]. Some of the potential clinical advantages of Flurpiridaz F-18 are unit dose availability, high image resolution, high extraction fraction and perfusion defect resolution, low radiation exposure, feasibility of rest-treadmill exercise imaging, peak stress function imaging, and most importantly, diagnostic accuracy for detection of CAD[6]. In comparison to SPECT MPI, the most common procedure used in nuclear cardiology today, Flurpiridaz PET has higher diagnostic efficacy and image quality in patients with suspected CAD, as demonstrated by the announcement made by GE Healthcare and Lantheus Holdings Inc. at the American Society of Nuclear Cardiology Congress[7].

## Comparative analysis of flurpiridaz along existing radiotracers

In nuclear cardiology, various radiotracers are employed in PET and SPECT modalities. For PET, these include 82Rb, 13 N-ammonia, and 15O-water, while SPECT commonly utilizes 99mTc-based agents such as sestamibi and tetrofosmin. Most of these tracers are clinically beneficial, but with limitations. PET tracers (such as, 82Rb) are generator based, but have a very short half-life (75 s) and low spatial resolution due to the high positron energy, which limits image quality[8]. The PET tracers 13 N-ammonia and 15O-water provide wonderful diagnostic information, but they demand an on-site cyclotron and limit their broad approval in hospital plans. SPECT tracers are more practically approachable; however, they suffer from lower spatial resolution, attenuating artifacts, and eventually limit the user’s capability to measure myocardial blood flow[8].

Flurpiridaz has distinct advantages over existing radiotracers. It provides the high spatial resolution characteristic of PET imaging and offers a longer physical half-life (about 110 min), which allows for centralized production and wider distribution^[3,5]^. Unlike traditional PET tracers, Flurpiridaz is appropriate for both exercise and pharmacological stress testing, providing solid clinical flexibility[5]. In addition, with a first-pass myocardial extraction fraction >90%, Flurpiridaz PET imaging will provide a better quality image, and it will also allow greater accuracy in quantifying myocardial blood flow[5]. Clinical trials, namely the AURORA Phase III study, establish that Flurpiridaz PET imaging had higher sensitivity (80.3%) and non-inferior specificity (63.8%) than SPECT, with a particularly improved performance in women and obese patients[3].

With the Food and Drug Administration (FDA) approval for Flurpiridaz, the gaps in the nuclear cardiac imaging landscape have been filled by providing a better approach to high-quality PET imaging without the disadvantages that were set by short-lived tracers or cyclotron availability[5]. Additionally, with the greater resolution and defect characterization that Flurpiridaz provides, diagnostic certainty may be improved in patient populations that are often not well addressed by SPECT^[5,8]^. Overall, Flurpiridaz moves the practice of cardiac imaging beyond the practical limitations of current radiotracers and is a meaningful progress in our field.

## Significance of flurpiridaz FDA’s approval

### Pre-approval challenges

The Flurpiridaz approval process faced many obstacles. Early phase III trials, although demonstrating higher sensitivity compared with SPECT modalities, did not achieve sufficient relative specificity, prompting the initiation of a second phase III study: the AURORA trial. Flurpiridaz also faced the challenge of competing with settled tracers, including 99mTc-based SPECT agents, which have decades of established use and utility, and support from health care systems[5]. Flurpiridaz was further limited in its use as an investigational agent, which restricted its availability, thereby limiting access and experience for patients and clinicians. Lastly, gaining approval required strong evidence to explain that Flurpiridaz was evenly effective, safe, useful, and basically beneficial to clinical care^[3,5]^.

### Post-approval benefits

The FDA’s approval of Flurpiridaz signifies a highly important step in nuclear cardiology. Clinically, Flurpiridaz is expected to produce better patient outcomes by facilitating the earlier diagnosis of CAD with its high spatial determination and myocardial extraction fraction, which improve the recognition of subtle perfusion abnormalities, particularly with women and obese patients[3].

We anticipate the approval to ease wider clinical adoption of PET MPI. Flurpiridaz has a longer half-life that supports regional cyclotron production and distribution, allowing institutions without a cyclotron to provide quality PET MPI[5]. Approval of a drug is meaningless without an infrastructure in place that accounts for the addition of scanners and staff trained in PET[5].

Overall, while PET imaging will have a higher economic cost initially than SPECT, the better diagnostic performance of Flurpiridaz will likely decrease the overall number of studies done, limit the number of invasive procedures taken, and qualify earlier therapeutic interventions, eventually lowering health costs^[3,5]^. Early diagnosis of CAD using Flurpiridaz may also limit disease progression and provide overall savings for health environments.

### Safety and efficacy

Researchers studied dosimetry, biodistribution, and safety of Flurpiridaz following a single-dose injection at rest. The kidneys received the highest average absorbed dose, followed closely by the heart wall. Remarkably, high and sustained retention of radioactivity in the heart was observed from the earliest images and lasted for about 5 h post-injection. There were no drug-related adverse events, and the tracer was well tolerated by all participants[9].

Among the adverse events reported within 2 weeks of receiving Flurpiridaz, 70% of the subjects reported such events. Most of these were linked to underlying medical conditions, the use of pharmacological stressors, treadmill exercise, or subsequent coronary interventions. Around 7.4% of the adverse events reported were possibly related to Flurpiridaz, but none were deemed serious. Importantly, there were no significant adverse laboratory changes or clinically relevant electrocardiographic changes observed after the injection[9]. Clinical studies have confirmed that Flurpiridaz PET is safe for use[9].

Dosing for Flurpiridaz was carefully specified as 2.5–3.0 mCi for rest, 9.0–9.5 mCi for exercise (with at least a 1-h interval between rest and exercise doses), and 6.0–6.5 mCi for pharmacological stress (with a minimum 30-min interval)[9].

Current guidelines advocate for dose reduction strategies, which means that over 50% of patients undergoing SPECT-MPI can receive a total effective dose of less than 9 mSv. Despite these lower doses, 89% of studies reported good to excellent image quality. In fact, the average radiation dose of Flurpiridaz, which is about 6.1 mSv, could be further minimized to 2.9 mSv with optimized acquisition and processing. The dose could drop even lower to 0.6 mSv, if a stress-only protocol is used[9].

Flurpiridaz PET demonstrated significantly higher sensitivities and non-inferior specificities compared with SPECT. By the majority rule, it also showed significantly better sensitivity for detecting CAD, while its specificity remained comparable to that of SPECT[3]. It also successfully met the primary efficacy endpoint by demonstrating sensitivity and specificity for detecting CAD that significantly surpassed predefined criteria[3].

### Future implications

Flurpiridaz F-18 has an important catalytic function in nuclear cardiology. It is a driving force behind the expansion of nuclear perfusion imaging and the creation of new avenues for improved CAD diagnosis, risk assessment, and treatment[10]. Early detection of heart disease can also assist in avoiding catastrophic complications like heart attacks, strokes, and heart failure. Preventive efforts can be implemented to avoid these potentially catastrophic episodes if illness is detected early on[11].

This innovative drug excelled in terms of image quality, interpretative confidence, and reduced radiation dose to patients. With its higher image resolution, improved myocardial extraction fraction, potential for single-dose delivery, and compatibility with treadmill exercise, Flurpiridaz may pave the way for broader adoption of PET imaging. Its extended half-life of about 110 min enables its use in hospitals without onsite cyclotron production to offer high-quality PET MPI[3]. By reducing the need for invasive treatments and facilitating earlier, accurate detection, noninvasive diagnostic techniques for CAD can enhance patient outcomes and save health care costs[12].

### Challenges

Adopting Flurpiridaz (F-18) presents several challenges related to cost, access, training, and availability of PET scanners. The high costs associated with PET scanners and the establishment of cyclotron facilities can be prohibitive, especially in low- and middle-income countries. Although the longer half-life of Flurpiridaz reduces the need for onsite cyclotrons, regulatory approvals, in Brazil for example, can be a significant obstacle in its implementation[13].

Additionally, limited access to PET scanners in some regions further restricts its use. The introduction of Flurpiridaz also requires specialized training for health care professionals and technologists alike, further delaying widespread adoption. The introduction of comprehensive training programs can be costly and time-consuming, particularly in regions with limited medical infrastructure[14].

## Conclusion

Hence, the FDA approval of Flurpiridaz F-18 showcases a revolutionary moment that addresses an existing gap in nuclear cardiology imaging and provides a flexible, high-resolution, and accessible imaging modality with the potential to considerably transform the landscape of cardiac care. Superior diagnostic accuracy, enhanced patient safety, and wider availability through centralized production make Flurpiridaz capable of overcoming many limitations associated with traditional tracers. Despite cost, infrastructure, and training challenges – especially in resource-limited settings – this discovery paves the way for earlier CAD detection, better management, and improved long-term outcomes.

## Data Availability

No datasets were generated or analyzed during the current study.
